# A Cross-Sectional Study of the Association between Autoantibodies and Qualitative Ultrasound Index of Bone in an Elderly Sample without Clinical Autoimmune Disease

**DOI:** 10.1155/2018/9407971

**Published:** 2018-04-30

**Authors:** Rosebella A. Iseme, Mark McEvoy, Brian Kelly, Linda Agnew, Frederick R. Walker, Michael Boyle, John Attia

**Affiliations:** ^1^Department of Population and Reproductive Health, School of Public Health, Kenyatta University, P.O. Box 43844, Nairobi 00100, Kenya; ^2^School of Medicine & Public Health, The University of Newcastle, Callaghan, NSW 2308, Australia; ^3^Hunter Medical Research Institute, Kookaburra Circuit, New Lambton Heights, NSW 2305, Australia; ^4^Centre for Brain and Mental Health Research, The University of Newcastle, Callaghan, NSW, Australia; ^5^Brain Behaviour Research Group, School of Science and Technology, University of New England, Armidale, NSW 2351, Australia; ^6^Laboratory of Affective Neuroscience, The University of Newcastle, Callaghan, NSW, Australia; ^7^University of Newcastle, Medical Sciences MS413, University Drive, Callaghan, NSW 2308, Australia; ^8^Department of General Medicine, John Hunter Hospital, New Lambton Heights, NSW, Australia

## Abstract

Bone loss is characteristic of the ageing process and a common complication of many autoimmune diseases. Research has highlighted a potential role of autoantibodies in pathologic bone loss. The confounding effects of immunomodulatory drugs make it difficult to establish the contribution of autoantibodies amongst autoimmune disease sufferers. We attempted to examine the relationship between autoantibodies and bone mass in a population of 2812 elderly participants without clinical autoimmune disease. Serum samples were assayed for a panel of autoantibodies (anti-nuclear, extractable nuclear antigen, anti-neutrophil cytoplasmic, thyroid peroxidase, tissue transglutaminase, anti-cardiolipin, rheumatoid factor, and cyclic citrullinated peptide). Bone mass was measured using quantitative ultrasound (QUS) of the calcaneus. The relationship between each autoantibody and bone mass was determined using linear regression models. Anti-nuclear autoantibodies were the most prevalent, positive in approximately 11%, and borderline in roughly 23% of our sample. They were also the only autoantibody observed to be significantly associated with QUS index in the univariate analysis (*n* = 1628; *r* = −0.20; 95% CI: −0.40–0.00; *p* = 0.046). However, statistical significance was lost after adjustment for various other potential confounders. None of the other autoantibodies was associated with QUS index in either univariate or multivariate analysis. We are limited by the cross-sectional nature of the study and the low prevalence of autoantibodies in our nonclinical sample.

## 1. Introduction

Normal bone remodelling requires a tight coupling of bone resorption to bone formation, whereby the two processes occur simultaneously and in harmony to guarantee negligible change in bone mass and therefore no alteration in bone quantity after each remodelling [1]. Loss of coupling between these two processes leads to osteoporosis, a combination of pathologic bone loss and altered microarchitecture that results in fragility fractures in response to minimal or low velocity force [2, 3]. Osteoporosis is predominantly a condition of the elderly and accounts for approximately 2 million fractures annually, including hip, vertebral (spinal), wrist, and other fractures [3, 4]. Osteoporotic fractures contribute to a marked increase in morbidity and mortality, as well as health care costs and disability amongst this cohort [3].

In recent years, pathologic bone loss has been associated with disorders characterised by immune dysfunction, hinting at the presence of an immune-skeletal interface [5, 6]. Research aimed at elaborating this relationship has identified common cell types and shared mediators that play functional roles in both systems [7]. For example, vitamin D, parathyroid hormone (PTH), testosterone, and leptin, all recognized regulators of bone function, are also acknowledged to modulate immune function [8]. Additionally, macrophages, osteoclasts, and dendritic cells are all derived from the same myeloid precursors, with the latter two noted to exhibit the same lifecycle [9]. Notably, varieties of immune cells have been observed to regulate osteoclast and osteoblast activity in turn mediating the process of bone remodelling, responsible for maintaining the quality of the skeleton [10]. It therefore stands to reason that perturbations in the immune system would translate into disruptions in bone homeostasis.

The emergence of the field of osteoimmunology has recognized the immune system as a vital player in fine-tuning the balance between bone resorption and bone formation [11]. Though the role of inflammatory cytokines such as tumor necrosis factor alpha (TNF*α*), interleukin- (IL-) 1*β*, and IL-6 in enhancing osteoclast-mediated bone resorption is well established, the involvement of autoantibodies is still poorly defined [12]. Nevertheless, the findings of our recent literature review provided strong evidence justifying further research aimed at delineating the relationship between autoantibodies and bone mineral density (BMD) [13]. Autoantibodies against a myriad of antigens have been linked to pathologic bone loss [14–20]. For instance, autoantibodies against rheumatoid factor (RHF) and anti-cyclic citrullinated peptide autoantibodies (anti-CCP) have been identified as independent risk factors for the development of bone erosion and osteoporosis in rheumatoid arthritis (RA) [14–17]. Similarly, autoantibodies targeting tissue transglutaminase (anti-TTG) have been significantly associated with a higher risk of hip fracture and reduced BMD levels in celiac disease [18, 19]. Moreover, anti-nuclear autoantibodies (ANAs) have been implicated in reduced BMD in systemic lupus erythematosus (SLE) populations [20]. These findings were however not confirmed by all studies [21, 22].

Notably, many of the studies attempting to characterise the relationship between autoantibodies and bone mass have been conducted in clinical populations with autoimmune conditions [15–28], where there are confounding effects of immunomodulatory drugs such as steroids, which are themselves implicated in pathologic bone loss. Studies examining the usefulness of autoantibodies as a method of screening the general population for osteoporosis are few and contradictory. Our literature review identified a single study utilising middle-aged women drawn from the general population that failed to verify previous findings that autoantibodies against osteoprotegerin (OPG) correlate with BMD [29]. Alternatively, studies comprising samples without any gastrointestinal symptoms of celiac disease, villous atrophy, or evidence of malabsorption reported significant associations between low BMD and increased circulating concentrations of autoantibodies against TTG and EMA (anti endomysial autoantibodies) [23–25]. Furthermore, the clinical utility of autoantibodies in relation to monitoring BMD variability remains an important research question on account of the increasing body of research. Studies utilising clinically autoimmune samples reporting observations of a significant association between a range of autoantibodies and BMD remain important observations given these associations remained significant even after adjusting for age, gender, body mass index (BMI), vitamin D, and smoking habits, all important mediators of bone mass [17]. Moreover, studies utilising early arthritis patients prior to the clinical onset of disease and before the start of treatment have further demonstrated a potential role for autoantibodies in mediating bone health through observations of significant associations between autoantibodies such as anti-CCP and anti-RF and decreased BMD independent of disease activity, specifically inflammatory status [26–28]. Furthermore, animal models have documented the ability of autoantibodies to directly upregulate bone resorption, providing compelling support for a causative role of these antibodies in pathologic bone loss [30–32].

Quantitative ultrasound (QUS) is a noninvasive ultrasound-based technique useful in assessing bone structure (elasticity and microarchitecture) as well as bone mass, therefore providing determinations of bone quality beyond those associated with the dual-energy X-ray absorptiometry (DEXA) [33]. The latter technique is currently considered the gold standard and comprises an accurate technique used to measure BMD at specific fracture-related sites, namely, the spine, hip, and radius [33]. Recently, research has acknowledged the role of bone microarchitecture and elasticity in conjunction with bone density in the development of bone fragility and subsequent fractures, in turn highlighting the important contribution of the QUS measurements [34]. The widespread interest in QUS as a useful measurement tool is also increasingly attributed to it being a rapid, portable, and radiation-free technique making it a more suitable alternative to DEXA for screening a large sample of relatively healthy elderly subjects [35–42]. Additionally, QUS measurements of bone property have been illustrated to correlate with real and volumetric BMD measured by DEXA [37, 38, 40, 42–48]. In this study, we shall take advantage of heel QUS to assess bone properties of our healthy elderly sample.

Age is an important risk factor for autoimmunity, with several autoimmune diseases preferentially occurring more prevalently in the second half of adulthood [49, 50]. Moreover, several studies have reported a higher prevalence of both organ and non-organ-specific autoantibodies amongst the elderly even in the absence of autoimmune disease when compared to the general population [49]. Of particular interest is the fact that the elderly represent a population characterised by high levels of autoantibodies that do not necessarily reflect clinical autoimmune disease [50]. We were therefore able to take advantage of the latter characteristic to evaluate the association between autoantibodies and bone mass in a population without clinical autoimmune disease in order to eliminate the confounding effect of immunomodulatory drugs.

Osteoporosis is one of the most prominent worldwide public health problems, and morbidity is increasing with the ageing global population [51]. As a silent disease without obvious symptoms and evidence until fracture, early diagnosis remains the key strategy to enable efficient management of this condition. Research has successfully demonstrated the benefits associated with early diagnosis and subsequent intervention on the delayed progression of the disease as well as improved outcomes [52]. Therefore, searching out biomarkers that are able to identify individuals at high risk of developing osteoporosis particularly at a time when BMD measurements of DEXA or QUS do not offer enough information to make a diagnosis would prove particularly useful.

## 2. Materials and Methods

### 2.1. Ethical Statement

This research was approved by the Human Research Ethics Committees of the Hunter New England Health District and the University of Newcastle (03/12/10/3.26).

### 2.2. Population

Participants were drawn from the Hunter Community Study (HCS), a longitudinal study comprising a cohort of 3318 community-dwelling Australians aged 55–85 years, randomly selected from the New South Wales (NSW) electoral roll. The specifics of their recruitment and characteristics have previously been described [53].

Individuals with clinical autoimmune disorders were excluded from this analysis (Table 1). These exclusions were instituted to investigate the link between autoantibody and bone mass in a nonclinical sample in order to eliminate the confounding effect of immunomodulatory medications associated with pathologic bone loss. Study participants with osteoporosis were additionally excluded from this analysis.

### 2.3. Data Collection

At baseline, study participants completed a range of postal questionnaires covering a wide range of data such as medical history, smoking habits, medication use, and dietary habits. Self-report questionnaires were returned by participants when they attended the HCS data collection clinic during which time blood samples were collected and included plasma, serum, whole blood, and DNA that was stored at 80 degrees Celsius in 1 mL aliquots to minimize freeze-thaw cycles. Additional clinical measures obtained at the data collection center included BMI, level of physical activity, and QUS measurements. Study participants also consented to the linkage of their HCS study data to local and national information databases and records (Table 2). Linking of HCS data to these databases provided a range of detailed information including data on the use of prescription medication.

### 2.4. Autoantibody Measurements

Serum autoantibody titres were determined using a variety of assays.

- HEp-2 ANA slides supplied by Kallestad (Bio-Rad Laboratories) were used to measure anti-nuclear autoantibody (ANA) titre. Negative, borderline, and positive categories corresponded to titres < 1 : 40, 1 : 40 ≥ titres ≤1 : 80, and titres ≥ 1 : 160, respectively.
  - Individuals classified as borderline or positive for ANA were subsequently tested for extractable nuclear antigen antibodies (ENA) using an enzyme-linked immunosorbent assay (ELISA). Samples were screened for 6 antigens, namely, anti-Smith (Sm), ribonucleoprotein (RNP), Sjogren's syndrome A and B (SSA and SSB), topoisomerase I (SCL-70), and autoantibodies against amino acyl-tRNA synthetases (Jo-1) (ImmunoConcepts, USA). Individuals who tested positive for ENA but had no defined antigen specificity identified were classified as borderline for ENA, whilst those who had at least one of the six antibody specificities identified were classified as positive.
- Commercial formalin-fixed neutrophil slides (INOVA Diagnostics Inc., San Diego, California) were used to measure anti-neutrophil cytoplasmic antibodies (ANCA). Indeterminate and atypical ANCA was categorised as borderline whilst those staining with a cytoplasmic fluorescence of classical cytoplasmic or perinuclear pattern of 1 : 10 or higher dilution were classified as positive.
- ELISA (Aesku, Germany) was used to test for thyroid peroxidase (TPO) autoantibody titres. Titres ≥ 50 units per millilitre (units/mL) were deemed positive.
- AESKULISA CeliCheck immunoglobulin A (IgA) and immunoglobulin G (IgG) tissue transglutaminase (TTG) ELISA (six-point calibrator) was used to measure TTG autoantibody titres where titres ≥ 25 units/mL were considered positive.
- ELISA produced by Medical Innovations (four-point calibrator curve) was used to test for anti-cardiolipin antibodies (ACGA). Titres ≤ 5 IgG phospholipid units (GPL) were defined as negative whilst 6–20 GPL was low positive, 21–40 GPL moderate positive, and over 40 GPL high positive.
- Rheumatoid factor (RHF) was measured using the RHF Beckman Coulter Immage Immunochemistry system, and titres ≥ 20 international unit per millilitre (IU/mL) were defined as positive.
- Anti-cyclic citrullinated peptide antibodies (anti-CCP) were measured using an anti-CCP2 kit QUANTA lite (INOVA Diagnostics Inc., San Diego) with titres > 20 enzyme immunoassay units per millilitre (EU/mL) defined as positive.

All ELISA were performed on the Grifols Triturus platform (Grifols USA, LLC).

### 2.5. Outcome Measure

Bone mass was measured at the heel using quantitative ultrasound (QUS) (Sahara Hologic sonometer, Hologic Inc., MA, USA). The calcaneus (heel bone) is a recognized and preferred peripheral site for assessing bone quality because it has high metabolic turnover rate and possesses two lateral surfaces, which facilitate the movement of ultrasound through the bone [54]. The heel bone also contains a large percentage of trabecular bone (~95%), which has a high metabolic turnover and a pattern of bone loss similar to the spine [55–57]. Results were expressed as QUS index. The QUS index is a composite parameter derived from the two basic measurements generated by the QUS, that is, the speed of sound (SOS) and the broadband ultrasound attenuation (BUA). Research has shown the QUS index (QUI) to be a more useful determinant of bone health status capable of differentiating subjects with a history of fractures as well as predicting future fracture risk in both men and women as well as discriminating women with low BMD from healthy postmenopausal women [43–46]. In addition, several studies have also reported a strong correlation between QUI obtained from QUS and DEXA measurements [58–60]. The QUS index assesses both dimensional structure and bone strength and therefore has a range of clinical applications.

The instrument was calibrated every morning using a phantom, according to the manufacturer's recommendations to protect the long-term stability of the measurement tool.

### 2.6. Confounders

**Figure 1:**
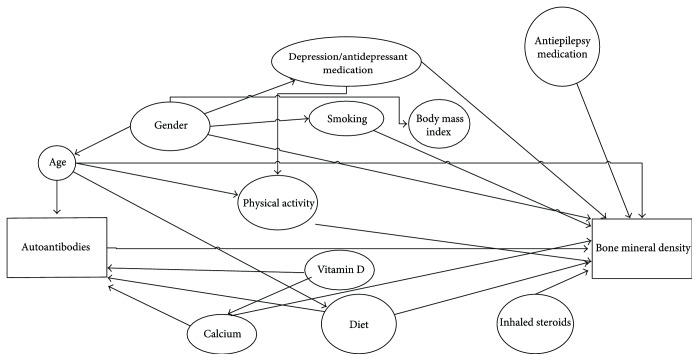
Directed acyclic graphs: determining confounding variables.

Potential confounders were purposefully selected using directed acyclic graphs (DAGs), in combination with discussions with content experts (Figure 1) [61]. These included demographic data (age and gender), clinical measures (BMI), lifestyle factors (smoking status, diet, and physical activity), the use of medications likely to be associated with bone metabolism or risk of falls (antiepileptics, antidepressants, and inhaled steroids), and the use of vitamin D and calcium in the form of supplements. Vitamin D and calcium are recognized regulators of bone homeostasis [62].

Data on demographic and lifestyle factors such as age, gender, and smoking habits were collected via self-report questionnaires. Data on medications including the use of vitamin D and calcium was also collected via self-report questionnaires and additionally via linkage to Medicare Australia and Pharmaceutical Benefits Scheme (PBS) that enabled collection of information on prescription drug use. BMI (weight/height^2^; kg/m^2^) was calculated during the clinical visit using height readings measured with a seca wall-mounted stadiometer and weight readings measured with a digital scale.

Physical activity (PA) was measured using step count. Study participants were required to wear a pedometer for seven consecutive days during waking hours to enable mean daily steps to be calculated. Additionally, nutritional assessment was carried out using the Australian Recommended Food Score (ARFS) [63]. The ARFS was calculated based on national recommendations in the Dietary Guidelines for Australian Adults and the core foods given in the Australian Guide to Healthy Eating (AGHE). Respondents are able to obtain a total of 74 points. As a result of missing data, HCS participants were only able to score a possible total Australian Recommended Food Score (TARFS) of 67 points. The scoring method is described in Table 3. A higher score is indicative of greater diet quality.

### 2.7. Statistics

The nature of the relationship between our selected autoantibodies and bone mass was determined using linear regression models. Four linear regression models were developed for each of our autoantibodies. The association between each autoimmune marker and QUS index was initially examined alone. Thereafter, we included other factors known to influence bone mineral density to each autoantibody model:

- Autoantibody alone (model 1)
- Autoantibody alone run separately for male and female gender (model 2a and 2b)
- Autoantibody + age (model 3)
- Autoantibody + age + gender + smoking class + BMI + TARFS + vitamin D + calcium (model 4)
- Autoantibody + age + gender + smoking class + BMI + TARFS + vitamin D + calcium + physical activity + antidepressants + inhaled steroids + antiepileptics (model 5)

Given that there was no statistically significant association with either physical activity, antidepressants, inhaled steroids, or antiepileptic medication use and QUS index in univariate regression, these variables were only added in sensitivity analysis. We additionally examined the impact on bone of expressing more than one autoantibody. All analyses were performed in Stata software v11 [64]. Results are expressed as coefficients with corresponding 95% confidence intervals and *p* values. Significance was set at *p* < 0.05.

## 3. Results

### 3.1. Characteristics of Study Participants and Association with Bone Mineral Density

There was a total of 2812 study participants included in this study following the exclusion of persons with clinical autoimmune disease (*n* = 445) and osteoporosis (*n* = 61). Amongst these participants in the community-dwelling sample, 1246 (44%) were male, median age was 65 years, mean total Australian Recommended Food Score (ARFS) was approximately 28/67, and median step count was 6534.25 per day. Notably, the majority of our sample were overweight (*n* = 1151; 40.93%) and had never smoked (*n* = 1432; 50.92%). Additionally, 309 (11%) and 47 (2%) study participants were noted to be using calcium and vitamin D supplements, respectively, according to self report and linkage data. Other demographic and disease-related characteristics are presented in Table 4.

Table 5 describes the coefficients for the univariate linear regression analysis (using available cases) for our selected covariates in relation to QUS index. As expected, QUS index was significantly associated with age, gender, smoking, BMI, diet (TARFS), vitamin D, and calcium intake. The results indicated that QUS index was significantly different for males compared to their female counterparts, for individuals who had never smoked compared to past and current smokers, for individuals with a BMI of overweight and obese compared to those in the normal BMI category, and for individuals who were on vitamin D and calcium supplements compared to those who were not.

Linear regression established that a 1-year increase in age elicited a 0.01 decrease in QUS index (*p* = 0.006; 95% CI: −0.02–0.004). Males demonstrated a QUS index of 0.32 units higher than females (*p* = 0.000; 95% CI: 0.20–0.44). When compared to normal BMI, being underweight resulted in a QUS index of 0.59 units lower than individuals with a normal BMI (95% CI: −1.52–0.34; *p* = 0.215); however, this association failed to reach significance. Alternatively, a BMI of “overweight” and “obese” significantly increased QUS index by 0.33 and 0.48, respectively (*p* = 0.000; 95% CI: 0.17–0.49; 95% CI: 0.31–0.65, resp.). Similarly, past smokers had an increase in QUS index by 0.13 at borderline significance (*p* = 0.050; 95% CI: 0–0.26), whilst current smokers had a significantly decreased QUS index by 0.36 (*p* = 0.003; 95% CI: −0.60 to −0.12) compared to “never smokers.” Dietary intake was also positively correlated with QUS index (*r* = 0.01; 95% CI: 0.00–0.02; *p* = 0.034). Unusually, taking vitamin D and calcium supplements negatively correlated with QUS index (*r* = −0.59; 95% CI: −1.098 to −0.073; *p* = 0.025 and *r* = −0.35; 95% CI: −0.55 to −0.15; *p* = 0.001, resp.). Moreover, increasing physical activity by 1000 steps had a negligible effect on QUS index (*r* = 0.00; 95% CI: 0.00–0.00; *p* = 0.285). Also, whilst the use of antidepressants increased QUS index by 0.07, inhaled steroid use and antiepileptics decreased QUS index by 0.15 and 0.55, respectively. Neither of the latter medications were significantly associated with QUS index.

### 3.2. Autoantibody Prevalence

Autoantibody categories (borderline, positive, and negative) were defined based on healthy samples. Autoantibody prevalence varied across our sample. ANA prevalence was highest amongst our sample with approximately 17% (316/1850) found to be positive and 36% (669/1850) borderline for this autoantibody. More female study participants (172/850; 20%) were positive for ANA than their male counterparts (144/996; 14%). In 979 sera deemed to be positive or borderline for ANA that were subsequently tested for ENA, only 3% were positive with at least one of 6 specific ENA autoantibody specificities identified. Similar to ANA, more female study participants (17/501; 3%) were positive for ENA than their male counterparts (10/477; 2%). Autoantibodies to TTG, TPO, RHF, CCP, and ACGA were observed in 6% (119/1850), 9% (160/1848), 1% (19/1660), 4% (8/188), and 12% (223/1830) of our sample, respectively. Autoantibodies to CCP, TTG, and cardiolipin were the only ones to present more prevalently amongst males when compared to their female counterparts. Anti-RHF was the least prevalent autoantibody within our sample. Autoantibody prevalence is shown in Table 6.

### 3.3. Correlations between Quantitative Ultrasound Index (QUS Index) and Biochemical and Clinical Variables

The details of the linear regression analyses examining the association between autoantibodies and QUS index are presented in Tables 7 and 8. ANA positivity was negatively correlated with QUS index. The latter univariate association showed borderline significance (*r* = −0.20; 95% CI: −0.40–0.00; *p* = 0.046). After adjusting for age, the latter association was observed to be approaching significance; however, statistical significance further diminished with the addition of other covariates. After adjusting for age, those who were ANA positive had an average 0.19 lower QUS index than their negative counterparts (*p* = 0.058). Notably, as we moved from borderline to positive autoantibody categories, a larger decrease in QUS index is observed.

Although not reaching statistical significance, ENA, ANCA, TPO, TTG, CCP, and ACGA autoantibody positivity showed a similar tendency towards lower QUS index in the positive autoantibody categories compared to their negative counterparts. Anti-CCP autoantibodies elicited the largest decrease in QUS index; however, these immune markers were not significantly associated with QUS index (*r* = −0.43; 95% CI: −1.51–0.65; *p* = 0.437). Notably, anti-RHF positivity was observed to elicit an increase in QUS index compared to negative counterparts in the univariate analysis (*r* = 0.45; 95% CI: −0.44–1.34; *p* = 0.325). In general, the pattern across all autoimmune markers was similar; there was a larger effect in the univariate analysis which attenuated in the more adjusted models. Moreover, there was an apparent dose-response effect on QUS index in moving from negative to borderline to positive immune marker groups. Notably, examination of *R* squared (*R*^2^) illustrated that the addition of physical activity, antidepressants, inhaled steroids, and antiepileptics did not improve the model (model 5) (*R*^2^ data not shown).

A minority of our sample was positive for more than one autoantibody (Refer to Table 9). The specificities that largely overlapped amongst the coexpression of any two autoantibodies were ANA with TTG, TPO, ACGA, and ANCA as well as TPO and TTG, ACGA and ENA, TPO and TTG, plus ANCA and ENA, TPO, TTG, and ACGA. Sensitivity analyses examining the impact on bone being positive for more than one autoantibody yielded some significant results. Individuals who were positive for both ANCA and ACGA suffered a QUS index 0.84 less than their negative counterparts (95% CI: −1.53 to −0.15; *p* = 0.017) whilst individuals who were positive for TPO and TTG suffered a QUS index 0.85 less than their negative counterparts (95% CI: −1.63 to −0.06; *p* = 0.036). Even fewer individuals were positive for more than two autoantibodies, and the coexpression of three autoantibodies did not yield any significant correlation with QUS index.

## 4. Discussion

Despite existing literature pointing to a potential role of autoantibodies in modulating bone mass, it remains a relatively underresearched subject matter. Notably, the majority of existing research has investigated the relationship between autoantibodies and QUS using samples with clinical autoimmune disease [14–28]. Our results failed to observe any significant association between most of our autoimmune markers and QUS index. Nonetheless, this study provides novel data towards efforts aimed at ascertaining the potential role of autoantibodies in pathogenic bone loss. The results are particularly important, as our study comprised an elderly population sample with no clinical autoimmune disease, thus eliminating the influence of a range of immunomodulatory drugs on our observed outcomes. Moreover, we found only one study investigating the association between autoantibodies and bone utilizing QUS parameters amongst a healthy population sample [65]. Additionally, our findings also contribute to data examining the association between a range of anthropometric measurements and QUS index of the calcaneus amongst an elderly sample.

Although BMD obtained by DEXA is a standard diagnostic technique for osteoporosis, it is difficult to apply in community-based studies because of a lack of portability, high costs, and exposure to ionizing radiation [66]. At present, the QUS has generated widespread interest particularly as a population screening tool as it gives a quick evaluation of bone that is reportedly highly correlated with DEXA measurement of BMD, it is inexpensive and easy to carry, and it estimates the bone density of the calcaneus whilst also providing some information concerning the structural organization of the bone [66–69]. The QUS therefore improves accessibility to testing particularly amongst patients with restricted mobility.

Our results revealed a significant association between our QUS parameter and age, gender, BMI, smoking habits, and diet as well as vitamin D and calcium intake. These observations coincide with previous reports, where BMD was observed to be significantly associated with age, gender, BMI, and smoking habits [54, 70–72]. In fact, QUS variables have been acknowledged to decline with age similar to DEXA BMD measurements [73–75]. Our results confirmed the latter observation. Age-related bone loss is largely attributed to a rapid decline in sex hormones implicated in bone loss in varying amounts across both genders [71, 74, 76].

Gender as expected was positively associated with QUS index as you moved from female to male gender (*r* = 0.32; 95% CI: 0.20–0.44; *p* = 0.000). Gender-dependent differences in bone mass have been observed in both children and adults, with males reported to have a higher BMD than their female counterparts [77]. Similar gender-specific differences in QUS parameters have also been made evident by existing research [38, 54, 78, 79]. Gender-dependent differences in bone mass are linked to age-related decreases in sex hormones and differences in peak bone mass attained [74, 76]. Female gender is a well established risk factor for osteoporosis [80]. This is largely attributed to the role of sex hormones and sex hormone globulin that correlate with loss of BMD, fracture risk, and bone turnover [74, 76]. Additionally, gender differences in osteoporosis must be understood in the context of the physiology of bone maturation and skeletal growth as well as variations in anthropometric measures such as BMI between male and female genders [54, 80]. Briefly, males are acknowledged to achieve similar or higher bone density than females and at a later age [80]. Additionally, though gradual loss of bone mass is common across both genders with age, women tend to lose bone at a faster rate than their male counterparts [80]. Moreover, estrogen deficiency which plays a major role in osteoporosis development for both genders is noted to be more pronounced for women and begin at a younger age [74, 76, 80]. Furthermore, males are believed to have higher BMI than females and new research has also highlighted gender-related variations in molecular signaling between bone and muscle independent of purely mechanical interactions that result in gender differences in the acquisition and age-related loss in bone and muscle tissue [70, 71, 81]. Body weight is a known protective factor of bone loss [81]. As was depicted in our results, QUS index amongst individuals who are classified as overweight or obese is higher than those within the normal BMI category, *r* = 0.33; 95% CI: 0.17–0.49; *p* = 0.000 and *r* = 0.48; 95% CI: 0.31–0.65; *p* = 0.000, respectively. In this regard, a higher body weight is believed to lead to greater mechanical loading of bone with subsequent stimulation of bone formation and an increase in bone density [82]. Indeed, BMI has been reported to positively correlate with QUS index in postmenopausal women as well as in older male and female population samples [83, 84]. QUS index was lower amongst those classified as underweight when compared to normal BMI. This latter association however failed to reach statistical significance, an observation that is likely to have resulted from the small proportion of our sample that was underweight (*n* = 16).

Furthermore, our results coincide with reports of a recent meta-analysis that observed a significantly reduced bone mass amongst smokers compared with nonsmokers at all bone sites [72]. In fact, “current smokers” are observed to be a negative predictor of QUS parameters (BUA, SOS, and QUS index) amongst both men and women in previous studies [85]. Interestingly, autoantibodies have been proposed to mediate the effect of cigarette smoke on bone mass [13]. Notably, “smoking class ever” representing former smokers was significantly and positively associated with QUI in our elderly sample (*r* = 0.13; 95% CI: 0–0.26; *p* = 0.05). Previous studies using both the Sahara device and other sonometers have provided somewhat discrepant findings with both positive and negative associations reported between QUS parameters and previous smoking habits [86–90].

Diet was observed to positively correlate with QUS index amongst our elderly sample (*r* = 0.01; 95% CI: 0–0.02; *p* = 0.034). Our average TARF score (28 out of a possible 67 points) is suggestive of a diet that may not be consistent with consumption of a greater variety of foods as recommended by the Australian Dietary Guidelines [63]. Diet has been identified as an important mediator of osteoporosis risk [91]. In particular, excessive alcohol, caffeine, and tobacco use as well as low calcium and vitamin D are acknowledged to increase the risk of fragility fractures [92]. Inconsistent observations amongst studies investigating the association between diet and bone quality (mass and microarchitecture) in elderly samples have been attributed to differences in methods of measuring nutritional status (i.e., anthropometry and biochemical data versus anthropometry alone) as well as variations in study participant age [92]. Notably, a significant negative correlation was observed between calcium and vitamin D amongst our sample. Both calcium and vitamin D are recognized in existing literature as having a positive effect on bone health as they are important nutrients for the development, growth, and maintenance of a healthy skeleton throughout life [93, 94]. Vitamin D and calcium are closely linked through vitamin D's regulatory role of intestinal calcium absorption [93]. Contradictory reports exist describing the relationship between vitamin D, calcium, and bone mass [93–100]. According to existing literature, high dietary calcium intake and not daily calcium supplementation have been reported to enhance bone mass [94]. Moreover, the beneficial effect of calcium on BMD is reportedly only evident in physically active groups [95, 96]. Additionally, low dietary calcium intake has been linked to increased turnover of vitamin D metabolites, an observation that is proposed to affect the subsequent relationship between vitamin D and BMD [97]. Similarly, research has failed to illustrate the effectiveness of vitamin D supplements in increasing BMD [98]. However, a longitudinal study of institutionalized women illustrated a positive effect on quantitative ultrasound of bone of supplementation with vitamin D3 and calcium [100]. Notably, supplementation with vitamin D3 and calcium in the latter study highlighted that only BUA was observed to reflect the positive effect on bone of the latter nutrients [100]. As we lacked dietary vitamin D and calcium data, we were unable to clarify the relationship between these nutrients and QUS index in our study.

Our results failed to show a significant correlation between the use of antiepileptics, antidepressants, or inhaled steroids and QUI. Notably, contrary to previous research indicating that QUS parameters at the heel respond to physical activity, our results show that physical activity had no effect on QUS index [85, 97, 101]. Physical exercise is an acknowledged and important mediator of bone biomechanics [102]. Muscle contraction produces mechanical stress that results in activation of osteoblasts with subsequent bone formation [103]. Exercise is additionally recognized to promote bone mass acquisition through direct mechanical loading effects on bone in addition to muscle contraction [103]. Clear clinical guidelines regarding the most appropriate type, intensity, and duration of activity to prevent bone loss are however lacking. The prevailing general rule regarding exercise and BMD is that exercises that include loading, weight-bearing elements, and muscular strengthening factors are considered to be most appropriate in the context of osteoporosis [103]. Yet not all types of physical activity that provide bone loading to the skeleton have been shown to produce bone mass benefits [97]. There are also activities that provide bone loading at one site of the body but not at other sites [97]. This is based on the premise that osteogenic effects of exercise are specific to the anatomical sites where the mechanical strain occurs [97]. The calcaneus that plays a central position in supporting body weight is considered the skeletal site where maximal ground reaction forces are applied with every heel strike during exercise [94]. Nonetheless, according to a recent meta-analysis that sought to mathematically consolidate research on the effects of walking interventions on BMD in men and women aged 50 years and older, walking has a significant (*p* ≥ 0.03) positive effect on lumbar BMD but not femur or the calcaneus [104]. Moreover, studies that have examined the impact of physical activity on heel ultrasound are faulted for relying on historical self-report of physical activity [104]. On average, our sample walked 6534.25 steps per day. It is also possible that the amount of exercise undertaken by our study participants did not necessarily surpass the threshold necessary for modulating bone mass.

Interestingly, ANA was the most common autoimmune marker and the only autoantibody significantly associated with variability in QUS index in univariate analysis, an observation that was likely due to high power. The latter association was observed to be approaching significance after adjusting for age but disappeared following further adjustment for additional covariates. In particular, ANA was not significantly associated with QUS index when analysis was carried out separately for female and male genders (*r* = −0.18; 95% CI: −0.22–0.18; *p* = 0.857 and *r* = −0.13; 95% CI: −0.36–0.10; *p* = 0.267, resp.).

Existing literature has linked ANAs to lower BMD amongst cohorts with clinical autoimmune disease. In particular, anti-deoxyribonucleic acid (DNA) topoisomerase I autoantibodies have been noted to significantly correlate with BMD amongst a sample of Moroccan women with systemic sclerosis [105]. Additionally, anti-centromere autoantibodies have been identified as independent risk factors for bone damage amongst systemic sclerosis patients, whilst high anti-double-stranded DNA (dsDNA) autoantibody levels were observed to independently predict 10-year risk of incurring a hip fracture amongst SLE patients [20, 106]. ANAs represent one of the least researched immune markers in relation to pathologic bone loss. To our knowledge, this is the first study examining the association between ANAs and variability in bone mass in the absence of clinical autoimmunity. The production of ANAs is one of the major defining features of SLE, and their presence is part of the clinical diagnostic criteria [107]. Additionally, osteoporosis reportedly occurs in up to 68% of SLE sufferers [108]. The majority of studies examining the high prevalence of osteoporosis in SLE have however failed to explore the potential role of ANAs in mediating this relationship [108–111]. ANAs target a variety of nuclear antigens such as dsDNA, which are intimately involved in SLE pathogenesis [112]. Their exact mechanism of action in pathologic bone loss however remains unclear.

The direction and magnitude of the association between our autoimmune markers and QUS index were observed to be consistent. However, unlike ANA, the remaining autoantibodies did not reach statistical significance, likely due to the lower prevalence of these immune markers in our nondisease population. The dose-response effect was also reasonably consistent.

In particular, the small proportion of our sample positive for anti-ENA, anti-RHF, and anti-CCP autoantibodies significantly affected the power of our study and subsequently our ability to reliably estimate the association between the latter immune markers and QUS index, based on our results.

RHF was noted to have the lowest prevalence of all autoantibodies measured within our sample. The prevalence of these autoantibodies in the general population has been reported to increase with age [113, 114]. This increase is largely attributed to the effect of progressive senescence of immune function [114]. However, successfully ageing individuals (individuals lacking autoimmune or chronic disease) have been reported to have a prevalence of RHF which is not statistically significantly higher than a healthy young adult control group [115]. It is possible that the low RHF prevalence noted within our cohort was a result of the relatively good health of this sample.

It is important to note that previous research has illustrated a role for anti-RHF autoantibodies as enhancers of bone loss in the presence of anti-CCP autoantibodies [116]. However, a recent study, whereby radiographic progression in RA patients stratified by anti-CCP and RHF autoantibodies illustrated a more pronounced progression of structural damage associated with the presence of each autoantibody, contradicted these findings [19]. The latter study instead suggests an independent effect of RHF on bone loss in RA [19]. It has been proposed that the latter process is mediated by a proinflammatory environment resulting from activation of monocytes and macrophages through binding of RHF with low affinity Fc gamma (Fc*γ*) receptors found on their surface [13, 117, 118]. Furthermore, anti-CCP autoantibodies are acknowledged to bind to a diverse group of modified proteins in which arginine residues have been transformed into citrulline by peptidyl arginine deiminase [119]. However, not all their antigenic targets are implicated in modulating bone homeostasis [14, 120, 121]. At present, research has implicated citrullinated fibrinogen, enolase, and vimentin specificities in mediating bone loss via increased osteoclast resorption [14, 120, 121]. In particular, citrullinated vimentin receptors expressed on the surface of osteoclasts and myeloid precursors are highly implicated [14, 120–122]. The latter highlights the potential for these autoantibodies to stimulate differentiation of bone-resorbing osteoclasts, as well as trigger osteoclast-driven local bone resorption. Anti-CCP autoantibodies are implicated in early bone loss during the preclinical phase of RA and have been reported to independently predict bone erosion in RA patients independent of measures of disease activity such as the disease activity score for RA(DAS28) and inflammation as measured by levels of C-reactive protein (CRP) [121, 123].

Thyroid dysfunction is acknowledged as having unfavourable effects on the musculoskeletal system [124, 125]. Individuals with hyperthyroidism, subclinical hyperthyroidism, and hypothyroidism have been repeatedly observed to exhibit an increased fracture risk [124, 125]. In this regard, the aetiology of thyroid dysfunction is multifactorial and it remains unclear which underlying mechanisms are responsible for the comorbid osteoporosis. Subsequently, inconsistencies in studies linking fracture risk to the action of thyroid hormones versus thyroid autoantibodies means the effect of thyroid dysfunction on bone pathophysiology remains unclear [125, 126]. Notably, a recent cross-sectional population-based study examining the association between calcaneal ultrasound parameters and thyroid status in middle-aged and elderly Chinese men observed high anti-TPO levels (≥200 IU/mL) to be associated with lower QUI (*p* = 0.030) [65]. We failed to observe any significant association between anti-TPO autoantibodies and QUS index.

Previous literature has suggested a role for anti-TTG autoantibodies in bone disease observed to occur alongside conditions such as celiac disease, ankylosing spondylitis, and psoriatic arthritis [127, 128]. Moreover, anti-TTG autoantibodies have previously been shown to act as a marker of low BMD as well as high fracture frequency amongst an asymptomatic celiac disease population sample [18, 22]. Our study was unable to find any evidence of a statistically significant relationship between these autoantibodies and QUS index, likely due to low power. Anti-TTG autoantibodies are implicated in pathogenic bone loss through a variety of pathways. In particular, TTG has recently been identified as a regulator of receptor activator of nuclear factor kappa beta ligand (RANKL) production as well as myeloid and mesenchymal stem cell (MSC) differentiation [32]. The latter two cell types are the major precursors for osteoclasts and osteoblasts, respectively, whilst RANKL is the key factor for maturation, proliferation, and fusion of preosteoclasts as well as osteoclast activation and survival [13]. Inactivation of TTG by anti-TTG autoantibodies may therefore carry serious implications for bone homeostasis.

Anti-TTG and anti-TPO autoantibody cooccurrence was noted to significantly and negatively correlate with QUS index amongst our elderly sample (*r* = −0.85; 95% CI: −1.63 to −0.06; *p* = 0.036). Similarly, ANCA and ACGA autoantibody coexpression was also noted to significantly decrease QUS index in our sample (*r* = −0.84; 95% CI: −1.53 to −0.15; *p* = 0.017). Anti-TTG and anti-TPO autoantibodies have been reported in celiac disease sufferers who develop thyroid dysfunction [129]. Alternatively, ANCA and ACGA have been reported to cooccur in anti-neutrophil cytoplasmic autoantibody-associated diseases, primary sclerosing cholangitis and glomerulonephritis [130–132]. Unlike TPO and TTG, there is a lack of literature linking ANCA and ACGA to pathologic bone loss. Indeed, our observations may be an incidental abnormality as very few healthy individuals when screened would be expected to be positive for ANCA and ACGA. However, we cannot disregard the potential impact these autoantibodies, when coexpressed, might have on bone. Nonetheless, the significance of the coexpression of the latter autoantibodies in relation to bone fragility required further investigation.

Our study is not without its limitations. Firstly, the use of a nonclinical population sample significantly affected the prevalence of autoantibodies and therefore our ability to detect any clinically significant effect on QUS index. Furthermore, it was assumed that a vast majority of our female sample would already have entered menopause; therefore, this data was not collected and we were subsequently unable to control for its influence on the relationship between QUS index and autoantibody positivity. Moreover, our measurement of amount of physical activity rather than loading which is acknowledged to be a more important mediator of bone health may have affected our ability to accurately delineate the relationship between physical activity and QUS index. Similarly, our failure to account for dietary calcium and vitamin D intake may have also affected our ability to correctly describe the relationship between the latter nutrients and QUS index. Furthermore, we must acknowledge the potential for bias based on our use of self-report questionnaires. Additionally, our study is based on the assessment of an elderly Caucasian sample and therefore extrapolation of our findings beyond this group should be taken with caution. Nonetheless, the study is significantly strengthened by its use of standardised methods in the assessment of study characteristics amongst our sample drawn from the general population.

## 5. Conclusion

Existing research has linked high autoantibody titres to bone loss observed to occur alongside a variety of autoimmune diseases as well as present amongst the elderly. However, our study findings did not support the notion that autoantibodies are causative in bone disease. As previously mentioned, the use of a nonclinical population sample significantly affected the prevalence of autoantibodies and therefore our ability to detect any clinically significant effect on QUS index. Moreover, due to the cross-sectional nature of this study, this is purely explorative research. It would therefore be premature to conclude that autoantibodies have no impact on bone mass. Investigating the health impact of autoimmunity on bone health is important as it can point to latent or clinically silent forms of osteoporosis. Serological tests for autoimmunity could then be used to identify individuals with no or atypical symptoms at a time when QUS or DEXA are unable to provide any valuable information. The significance of autoantibodies in relation to bone health requires further investigation.

## Figures and Tables

**Table 1:** Autoimmune conditions and chronic diseases for exclusion.

*ANA related*
Lupus, SLE
Sjogren's syndrome
Scleroderma, CREST
Myositis: polymyositis, dermatomyositis
Autoimmune liver disease
Primary biliary cirrhosis
Polyarteritis nodosa
Cancer now (malignancy in last 5 years approximately)
Cancer ever (only melanoma included from skin cancers)
(Active) chronic infection (hepatitis A, hepatitis C)
*ANCA related*
Vasculitis
Wegener's
Churg-Strauss
Polyangiitis: microscopic polyangiitis, granulomatosis polyangiitis, eosinophilic granulomatosis with polyangiitis colitis
IBD: inflammatory bowel disease (not irritable bowel syndrome: IBS)
*ACGA related*
Antiphosholipid syndrome
Lupus inhibitor
*RHF/CCP-Ab related*
Rheumatoid arthritis
Psoriatic arthritis
Ankylosing spondylitis
*TTG-Ab related*
Celiac disease
*TPO-Ab related*
Thyroid disease: Grave's disease, Hashimoto's disease
*Chronic infections*
Glandular fever
Ross river virus
Malaria
Dengue

**Table 2:** Local and national health information databases linked to hunter community study data.

(i) Hunter New England Area Allied Health Records
(ii) Hunter New England Area Health Service Community Service Records
(iii) Hunter New England Area Health Service Outpatient Records
(iv) Hunter New England Area Health Service Hospital Admission Records
(v) Hunter New England Area Heart and Stroke Register and Diabetes Register
(vi) New South Wales Cancer Registry
(vii) Medicare Australia and Pharmaceutical Benefits Scheme

**Table 3:** Scoring method for foods listed in HCS DQESv2.

Food group	Items awarded 1 point	ARFS
Vegetables	>4 vegetables per day; potatoes cooked without fat; tomatoes fresh/canned; lettuce/endive/salad greens; carrots; cabbage/brussels sprouts; cauliflower; broccoli; celery; silver beet or spinach; peas; green beans; bean sprout or alfalfa sprout; pumpkin; onion or leeks; sweet corn; sweet potato; coleslaw; mushrooms; zucchini	20
Fruits	2 pieces of fruit/day; 1/week of each of fruit or vegetable juice; canned or frozen fruit; oranges or other citrus; apples; pears; bananas; melons (water, rock, honeydew); pineapple; strawberries; apricots; peach/nectarines; mango/pawpaw; avocado	14
Protein foods	Nuts; peanut butter or peanut paste; 1–4/week of each of beef, lamb, pork, chicken, fish (steamed, baked, or grilled fish/canned (salmon, tuna, sardines)); ≥1/week of each of baked beans, soya beans/soy beans and tofu; use up to 2 eggs per week	11
Grains	≥1/week of each of the following bread types with high fibre, wholemeal; ≥4 slices bread per day; ≥1/week All-Bran, Sultana bran, FibrePlus, Branflakes, Weetbix, VitaBrits, Weeties; rice; pasta/noodles	12
Dairy	Reduced fat or skim; 500 mL/day; cheese 1/week; ice-cream; yoghurt 1/week; use ricotta/cottage cheese; use low-fat cheese	7
Fats	Use nil/polyunsaturated/monounsaturated margarine	1
Alcohol	Drink beer/wine/spirits 1/month up to 4 days/week; or 2 glasses maximum/day	2

**Table 4:** Baseline demographic and disease-related characteristics of study population, total (*n* = 2812).

Descriptive characteristics	Median (IQR); *n* (%); mean (SD)
^∗^Age (years)—median (IQR)	65 (55–85)
Male gender—*n* (%)	1246 (44.31%)
^∗^PA (step count)—median (IQR)	6534.25 (4414.63–8572.75)
TARFS—mean (SD)	27.9627 (±8.02231)
Antidepressant—*n* (%)	257 (9.14%)
Inhaled steroids—*n* (%)	127 (4.52%)
Antiepileptics—*n* (%)	24 (0.85%)
Calcium—*n* (%)	309 (10.99%)
Vitamin D—*n* (%)	47 (1.67%)
(i) Smoking class “never”—*n* (%)	1432 (50.92%)
(ii) Smoking class “ever”—*n* (%)	1021 (36.31%)
(iii) Smoking class “now”—*n* (%)	211 (7.50%)
(a) BMI < 18.5 “underweight”—*n* (%)	16 (0.57%)
(b) 18.5 ≤ BMI< 25 “normal”—*n* (%)	548 (19.49%)
(c) 25 ≤ BMI< 30 “overweight”—*n* (%)	1151 (40.93%)
(d) BMI ≥ 30 “obese”—*n* (%)	863 (30.69%)

Categorical data is presented as frequencies and proportions in brackets. Where continuous data is not normally distributed data is presented as medians with interquartile ranges included in brackets. ∗ indicates data that is not normally distributed. Where data is normally distributed, means and standard deviations are presented. IQR: interquartile range; BMI: body mass index; PA: physical activity; TARFS: Total Australian Recommended Food Score.

**Table 5:** Effect of covariates on QUS index.

Variable	Coefficient	95% confidence interval	*p*
Age	−0.01	−0.02; −0.004	0.006
Gender	0.32	0.20; 0.44	0.000
PA (step count)	0.00	0.00; 0.00	0.285
TARFS	0.01	0.00; 0.02	0.034
Antidepressants	0.06	−0.15; 0.27	0.582
Inhaled steroids	−0.16	−0.47; 0.15	0.304
Antiepileptics	−0.57	−1.33; 0.19	0.145
Vitamin D	−0.59	−1.098; −0.073	0.025
Calcium	−0.35	−0.55; −0.15	0.001
Smoking class “ever”	0.13	0.00; 0.26	0.050
Smoking class “never”	Ref	—	—
Smoking class “now”	−0.36	−0.60; −0.12	0.003
BMI < 18.5 “underweight”	−0.59	−1.52; 0.34	0.215
18.5 ≤ BMI < 25 “normal”	Ref	—	—
25 ≤ BMI< 30 “overweight”	0.33	0.17; 0.49	0.000
BMI ≥ 30 “obese”	0.48	0.31; 0.65	0.000

PA: physical activity; TARFS: Total Australian Recommended Food Score.

**Table 6:** Autoantibody prevalence amongst study participants.

Autoantibody	Negative all	Females	Males	Positive all	Females	Males	Borderline all	Females	Males
Anti-nuclear autoantibodies (*n* = 1850; 850 females and 996 males)	865 (47%)	334 (40%)	518 (52%)	316 (17%)	172 (20%)	144 (14%)	669 (36%)	334 (39%)	334 (34%)
Extractable nuclear antigen autoantibodies (*n* = 979; 501 females and 477 males)	938 (96%)	477 (95%)	460 (96%)	27 (3%)	17 (3%)	10 (2%)	14 (1%)	7 (1%)	7 (1%)
Anti-neutrophil cytoplasmic autoantibodies (*n* = 1843; 844 females and 995 males)	1406 (76%)	620 (73%)	782 (79%)	145 (8%)	88 (10%)	57 (6%)	292 (16%)	136 (16%)	156 (16%)
Anti-cardiolipin immunoglobulin G autoantibodies (*n* = 1830; 840 females and 986 males)	1607 (88%)	742 (88%)	862 (87%)	223 (12%)	98 (12%)	124 (13%)	—	—	—
Rheumatoid factor autoantibodies (*n* = 1660; 767 females and 899 males)	1641 (99%)	745 (71%)	893 (78%)	19 (1%)	12 (1%)	6 (1%)	—	—	—
Tissue transglutaminase autoantibodies (*n* = 1850; 850 females and 996 males)	1731 (94%)	801 (70%)	926 (74%)	119 (6%)	49 (4%)	70 (6%)	—	—	—
Thyroid peroxidase autoantibodies (*n* = 1848; 850 females and 994 males)	1688 (91%)	752 (66%)	933 (75%)	160 (9%)	98 (9%)	61 (5%)	—	—	—
Anti-cyclic citrullinated peptide autoantibodies (*n* = 188; 93 females and 95 males)	180 (96%)	90 (8%)	90 (7%)	8 (4%)	3 (3%)	5 (5%)	—	—	—

Data is presented as frequencies with proportions included in brackets.

**Table 7:** Correlation between autoantibodies and quantitative ultrasound index (QUS index): univariate analysis.

Autoantibody	Model 1 (autoantibody alone) [coefficient; (95% CI); *p* value]	Model 2a (autoantibody alone, females only) [coefficient; (95% CI); *p* value]	Model 2b (autoantibody alone, males only) [coefficient; (95% CI); *p* value]
ANA *borderline*	−0.11	−0.18	−0.13
	(−0.26; 0.05)	(−0.22; 0.18)	(−0.36; 0.10)
	*p* = 0.168	*p* = 0.857	*p* = 0.267
	*n* = 1628	*n* = 749	*n* = 879

ANA *positive*	−0.20	−0.15	−0.16
	(−0.40; 0.00)	(−0.40; 0.09)	(−0.47; 0.15)
	*p* = 0.046	*p* = 0.228	*p* = 0.304
	*n* = 1628	*n* = 749	*n* = 879

Anti-ENA autoantibodies *borderline*	0.55	0.80	0.27
	(−0.38; 1.49)	(−0.25; 1.84)	(−1.38; 1.91)
	*p* = 0.245	*p* = 0.135	*p* = 0.749
	*n* = 877	*n* = 442	*n* = 435

Anti-ENA autoantibodies *positive*	−0.39	−0.21	−0.59
	(−0.99; 0.20)	(−0.86; 0.43)	(−1.70; 0.51)
	*p* = 0.194	*p* = 0.516	*p* = 0.291
	*n* = 877	*n* = 442	*n* = 435

ANCA *borderline*	−0.02	−0.04	0.01
	(−0.21; 0.18)	(−0.29; 0.21)	(−0.27; 0.30)
	*p* = 0.868	*p* = 0.744	*p* = 0.922
	*n* = 1621	*n* = 743	*n* = 878

ANCA *positive*	−0.19	0.12	−0.50
	(−0.45; 0.07)	(−0.18; 0.42)	(−0.93; −0.05)
	*p* = 0.160	*p* = 0.441	*p* = 0.028
	*n* = 1621	*n* = 743	*n* = 878

Anti-TPO autoantibodies *positive*	−0.21	−0.18	−0.11
	(−0.46; 0.04)	(−0.47; 0.10)	(−0.55; 0.33)
	*p* = 0.100	*p* = 0.201	*p* = 0.618
	*n* = 2114	*n* = 1011	*n* = 1099

Anti-RHF autoantibodies *positive*	0.45	0.31	0.76
	(−0.44; 1.34)	(−0.68; 1.30)	(−0.80; 2.32)
	*p* = 0.325	*p* = 0.536	*p* = 0.338
	*n* = 1947	*n* = 931	*n* = 1012

Anti-TTG autoantibodies *positive*	−0.15	−0.02	−0.28
	(−0.44; 0.14)	(−0.42; 0.37)	(−0.69; 0.14)
	*p* = 0.306	*p* = 0.902	*p* = 0.192
	*n* = 2114	*n* = 1011	*n* = 1099

Anti-CCP autoantibodies *positive*	−043	−0.39	−0.61
	(−1.51; 0.65)	(−2.11; 1.33)	(−2.03; 0.81)
	*p* = 0.437	*p* = 0.658	*p* = 0.400
	*n* = 2114	*n* = 1011	*n* = 1099

ACGA *positive*	−0.02	−0.10	−0.04
	(−0.23; 0.20)	(−0.38; 0.18)	(−0.27; 0.35)
	*p* = 0.887	*p* = 0.480	*p* = 0.804
	*n* = 1610	*n* = 740	*n* = 870

Data presented in bold represents significant results. Data presented in italics represents results approaching significance. 95% CI: 95% confidence interval; Ref: reference; ANA: anti-nuclear autoantibodies; Anti-ENA autoantibodies: anti-extractable nuclear antigen autoantibodies; ANCA: anti-neutrophil cytoplasmic autoantibodies; Anti-TPO: anti-thyroid peroxidase autoantibodies; Anti-RHF: anti-rheumatoid factor autoantibodies; Anti-TTG: anti-tissue transglutaminase autoantibodies; Anti-CCP: anti-cyclic citrullinated peptide autoantibodies; ACGA: anti-cardiolipin immunoglobulin G autoantibodies; BMI: body mass index; PA: physical activity; TARFS: Total Australian Recommended Food Score.

**Table 8:** Correlation between autoantibodies and quantitative ultrasound index (QUS index) after adjustment for potential confounders.

Autoantibody	Model 3 (autoantibody + age) [coefficient; (95% CI); *p* value]	Model 4 (autoantibody + age, gender, smoking class, BMI, TARFS, vitamin D, calcium) [coefficient; (95% CI); *p* value]	Model 5 (autoantibody + age, gender, smoking class, BMI, TARFS, vitamin D; calcium; antidepressants, inhaled steroids, antiepileptics; physical activity) [coefficient; (95% CI); *p* value]
ANA *borderline*	−0.10	−0.09	0.027
	(−0.25; 0.06)	(−0.25; 0.07)	(−0.17; 0.22)
	*p* = 0.217	*p* = 0.264	*p* = 0.784
	*n* = 1621	*n* = 1467	*n* = 1013

ANA *positive*	−0.19	−0.11	0.07
	(−0.39; 0.01)	(−0.32; 0.09)	(−0.18; 0.32)
	*p* = 0.058	*p* = 0.276	*p* = 0.586
	*n* = 1612	*n* = 1467	*n* = 1013

Anti-ENA autoantibodies *borderline*	0.52	0.70	0.75
	(−0.41; 1.45)	(−0.34; 1.73)	(−0.48; 1.98)
	*p* = 0.274	*p* = 0.186	*p* = 0.232
	*n* = 867	*n* = 790	*n* = 549

Anti-ENA autoantibodies *positive*	−0.39	−0.61	−0.95
	(−0.98; 0.21)	(−1.26; 0.03)	(−1.79; 0.11)
	*p* = 0.201	*p* = 0.062	*p* = 0.027
	*n* = 867	*n* = 790	*n* = 549

NCA *borderline*	−0.01	0.05	0.06
	(−0.21; 0.19)	(−0.15; 0.25)	(−0.19; 0.30)
	*p* = 0.911	*p* = 0.624	*p* = 0.648
	*n* = 1610	*n* = 1465	*n* = 1012

ANCA *positive*	−0.17	−0.10	0.12
	(−0.44; 0.07)	(−0.38; 0.17)	(−0.23; 0.47)
	*p* = 0.148	*p* = 0.461	*p* = 0.495
	*n* = 1610	*n* = 1465	*n* = 1012

Anti-TPO autoantibodies *positive*	−0.19	−0.15	0.035
	(−0.44; 0.07)	(−0.41; 0.11)	(−0.31; 0.38)
	*p* = 0.148	*p* = 0.257	*p* = 0.841
	*n* = 1804	*n* = 1640	*n* = 1144

Anti-RHF autoantibodies *positive*	−0.14	−0.11	−0.09
	(−0.35; 0.79)	(−0.34; 0.11)	(−0.35; 0.17)
	*p* = 0.214	*p* = 0.321	*p* = 0.497
	*n* = 1637	*n* = 1486	*n* = 1040

Anti-TTG autoantibodies *positive*	−0.15	−0.21	−0.23
	(−0.44; 0.14)	(−0.50; 0.09)	(−0.60; 0.15)
	*p* = 0.319	*p* = 0.166	*p* = 0.232
	*n* = 1804	*n* = 1640	*n* = 1144

Anti-CCP autoantibodies *positive*	−0.45	−0.65	−1.36
	(−1.52; 0.63)	(−1.79; 0.49)	(−2.76; 0.04)
	*p* = 0.415	*p* = 0.264	*p* = 0.058
	*n* = 1.804	*n* = 1640	*n* = 1144

ACGA *positive*	−0.02	−0.05	−0.098
	(−0.24; 0.19)	(−0.27; 0.17)	(−0.35; 0.16)
	*p* = 0.829	*p* = 0.674	*p* = 0.450
	*n* = 1599	*n* = 1454	*n* = 1005

Data presented in bold represents significant results. Data presented in italics represents results approaching significance. 95% CI: 95% confidence interval; Ref: reference; ANA: anti-nuclear autoantibodies; Anti-ENA autoantibodies: anti-extractable nuclear antigen autoantibodies; ANCA: anti-neutrophil cytoplasmic autoantibodies; Anti-TPO: anti-thyroid peroxidase autoantibodies; Anti-RHF: anti-rheumatoid factor autoantibodies; Anti-TTG: anti-tissue transglutaminase autoantibodies; Anti-CCP: anti-cyclic citrullinated peptide autoantibodies; ACGA: anti-cardiolipin immunoglobulin G autoantibodies; BMI: body mass index; PA: physical activity; TARFS: Total Australian Recommended Food Score.

**Table 9:** Association between quantitative ultrasound index (QUS index) and the coexpression of more than one autoantibody (for available cases).

Autoantibody	Correlation coefficient	95% confidence interval	*p* value	*N* pos
ANA + ENA	−0.67	−1.57; 0.23	0.144	10/1625 (1%)
ANA + ANCA	−0.12	−0.48; 0.23	0.497	72/1628 (4%)
ANA + TPO	−0.30	−0.79; 0.19	0.227	40/1628 (2%)
ANA + RHF	1.11	−0.53; 2.75	0.183	5/1589 (7%)
ANA + TTG	−0.35	−0.99; 0.29	0.283	24/1628 (1%)
ANA + ACGA	−0.04	−0.48; 0.39	0.850	46/1626 (3%)
ANCA + ENA	−0.22	−1.50; 1.05	0.729	5/1619 (0.3%)
ANCA + TTG	0.23	−0.78; 1.23	0.655	10/1628 (1%)
ANCA + ACGA	**−0.84**	**−1.53; −0.15**	**0.017**	**18/1619 (1%)**
ANCA + TPO	−0.38	−1.07; 0.31	0.283	21/1627 (1%)
ANCA + RHF	1.81	−1.03; 4.65	0.211	2/1609 (0.1%)
TPO + TTG	**−0.85**	**−1.63; −0.06**	**0.036**	**15/1628 (1%)**
TPO + ACGA	0.17	−0.52; 0.87	0.621	21/1625 (1%)
ANA + ANCA + TPO	−0.64	−1.50; 0.21	0.141	12/1628 (1%)
ANA + TPO + ACGA	−0.37	−1.53; 0.79	0.533	7/1627 (0.4%)
